# Supplementary material for: An Exploratory Investigation of Organic Chemicals Detected in Baby Teeth: Differences in Children with and without Autism
Source: J Xenobiot. 2024 Mar 14;14(1):404–15. doi: 10.3390/jox14010025 (PMC10971289; doi:10.3390/jox14010025)
Supplement: Supplementary file 1 [file jox-14-00025-s001.zip › jox-2800305-supplementary.pdf]

Supplementary Materials: An Exploratory Investigation of Organic Chemicals Detected in Baby Teeth: Differences in Children with and without Autism

Raymond F. Palmer

Table S1. Chemical function and product usage.

Variable.		
Chem1	Phthalate Isomer: 4, 5 and 7<br>CAS: 0-00-0	Androgen antagonist that suppresses the action of male sex hormones (e.g., testosterone), endocrine disrupting chemicals. These **phthalates** are part of the family of compounds used extensively in medical, automotive, and cosmetics production. **plasticizers** for PVC paste, pulp mixtures and as an additive in processing various products including soles for shoes, synthetic leather, waterproof membranes, paints, varnishes, floor coverings, door mats and hoses.
Chem2	Phthalate Isomer:5<br>CAS: 0-00-0	
Chem3	Phthalate Isomer:4<br>CAS: 0-00-0	
Chem4	Tris(2,4-di-tert-butylphenyl) phosphate<br>CAS: 95906-11-9	Organophosphorous compound for processing stabilizers for polymers. Flame retardant, **plasticizer**, lubricant additive, synthesis for rubber, coatings, and adhesives.
Chem5	tri(2-Ethylhexyl) trimellitate<br>CAS: 3319-31-1	PVC **plasticizer** for wire and cable insulation and interior automotive.
Chem6	Tonalid<br>CAS: 21145-77-7	Synthetic musk **fragrant**, chemical constituent of personal care products, enters body through dermal contact.
Chem7	Salicylic acid, 2-methylpropyl ether, 2-methylpropyl ester<br>CAS: 0-00-0	Skin medication for acme or wart treatment. Food additives containing phenols/salicylates are problematic for a subset of children and people with autism. Intolerance to Salicylate is linked to attentional problems, hyperactivity, mood and anxiety disorders-potentially through sulfation metabolism.
Chem8	Phenylmethanediol dibutanoate<br>CAS: 2929-77-3	Short-lived intermediate in some chemical reactions
Chem9	Phenol, 2,4-di-t-butyl-6-nitro-<br>CAS: 20039-94-5	Used as stabilizers, free-radical scavengers and antioxidants in technical applications, such as in fuels, hydraulic fluids and lubricating oils, as well as in elastomeric and thermoplastic **polymers**.
Chem10	Phenanthrene / Anthracene (co-elute) CAS: 85-01-8	Use to make dyes, **plastics, and pesticides**, explosives, and drugs.<br>Also found in particle emissions from natural gas combustion and municipal incinerator waste.<br>Particulates are present in ambient air pollution near high vehicular traffic and industrial or urban areas (ATSDR, 1995; Fang et al., 2006; Rehwagen et al., 2005).
Chem11	Oxime-, methoxy-phenyl-_<br>CAS: 0-00-0	anti-bacterial properties, found in melons, an aromatic
Chem12	Octyl methoxycinnamate<br>CAS: 5466-77-3	primarily used in sunscreens and other cosmetics to absorb UV-B rays from the sun, protecting the skin from damage.
Chem13	n-Hexyl salicylate<br>CAS: 6259-76-3	washing & cleaning products, air care products, polishes and waxes, cosmetics and personal care products and biocides (e.g. disinfectants, pest control products)
Chem14	Methyl dehydroabietate<br>CAS: 1235-74-1	An adhesive in food packaging. Viscosity control in emollients. Used in the preparation of **antimicrobial**s, cosmetics, skin conditioners,

Journal of Xenobiotics

MDPI

Chem15	Limonene<br>CAS: 5989-54-8	Major component in the oil of citrus fruit peels. a **flavoring**. Used in cosmetics, foods, cleaning products and **pesticides**. In foods, beverages, and chewing gum,
Chem16	Formamide, N,N-diethyl-<br>CAS: 617-84-5	An amide derived from formic acid, used as a chemical feedstock in the manufacture of pharmaceuticals, herbicides, **pesticides** and other chemical substances as an intermediate **solvent**. *Neurotoxin - Acute solvent syndrome*
Chem17	Formamide, N,N-dibutyl-<br>CAS: 761-65-9	
Chem18	Ethanol, 2-phenoxy-<br>CAS: 122-99-6	a **solvent** for dyes, inks, and resins; a lubricant preservative; and a reagent in organic chemical synthesis. a preservative for cosmetics and drugs, a fixative for perfumes to an attractant in **insecticides**. Exposure to phenoxyethanol has been linked to reactions ranging from eczema to severe, life-threatening allergic reactions. Infant oral exposure to phenoxyethanol can acutely affect nervous system function.
Chem19	DINP Isomer (Uncalibrated) Diisononyl phthalate ∣ CAS: 0-00-0	A **plasticizer**, especially in the production of polyvinyl chloride (PVC).
Chem20	Diisopropyl adipate ∣ CAS: 6938-94-9	A non-occlusive, non-oily, light emollient with excellent spreading properties. Used in bath oils, safe for use in cosmetics and personal care products.
Chem21	Cyclopentaneacetic acid, 3-oxo-2-pentyl-, methyl ester<br>CAS: 24851-98-7	Chemical intermediate. A kind of preparation method of natural fresh flower and fruit **flavor** essence.<br>Secondary alicyclic saturated and unsaturated alcohols, ketones and esters containing secondary alicyclic alcohols from phenol carboxylic acids
Chem22	Butyl benzoate<br>CAS: 136-60-7	perfume ingredient and as a **solvent** for cellulose ether, a dye carrier for textiles. It has a role as an **antimicrobial food preservative**,
Chem23	Benzothiazole (or 1,2-Benzisothiazole<br>CAS 272-16-2)<br>CAS: 95-16-9	pharmaceutical drug that possesses many attractive biological activities. It has **antibacterial** , anticonvulsant , anticancer , antifungal , antimitotic , and antitumor drugs Some benzothiazole derivatives are highly useful as **insecticides** and herbicides.
Chem24	Benzoic acid, 2-propenyl ester CAS: 583-04-0	Ingredient in perfumes and artificial fruit-**flavoring agents**.
Chem25	Benzeneacetaldehyde<br>CAS: 122-78-1	An intermediate in the manufacture of chemicals, **perfumes**, aniline dyes, **plastics**, synthetic rubber and in some fuel compounds.
Chem26	Benzamide, N-propyl-<br>CAS: 10546-70-0	Metabolite observed in cancer metabolism
Chem27	Benzaldehyde, 4-propyl-<br>CAS: 28785-06-0	For experimental research in food science **flavorings**
Chem28	Acetamide, N,N-dibutyl-<br>CAS: 1563-90-2	**Flavoring** agent or adjuvant.
Chem29	8-Hydroxycarvotanacetone<br>CAS: 7712-46-1	Extractant in essential oils
Chem30	4-Benzyloxybenzoic acid<br>CAS: 1486-51-7	An important class of **preservatives** extensively used in the cosmetic and pharmaceutical industries for preparing shampoos, commercial moisturizers, shaving gels, personal lubricants, topical/parenteral pharmaceuticals, spray tanning solutions, and toothpaste.
Chem31	2-Pyrrolidinone<br>CAS: 616-45-5	A variety of pharmaceutical drugs including cotinine, doxapram, povidone, and ethosuximide, and the racetams. Used in inkjet cartridges. *May damage the fetus.* Widely used organic polar solvent for various applications. 2-Pyrrolidinone is also an intermediate in the manufacture of polymers.<br>widely exists in various physiologically active natural products in nature. For example, it is the main structural unit of gonadotropin releasing

Journal of Xenobiotics

MDPI

		hormone. At the same time, 2-pyrrolidone is an important raw material and intermediate of medicine, pesticide, dye, peptide and other chemicals. If it is used as the end chain of peptide, it also plays a stable role in the conformation of the compound. Many polysubstituted 2-pyrrolidones have been used in the synthesis and production of a variety of drugs and applied for patents.
Chem32	2-Phenoxyethyl isobutyrate CAS: 103-60-6	**Flavoring and fragrant intermediary** agent and **solvent** for pharmaceuticals.
Chem33	2,4-Di-tert-butylphenol CAS: 96-76-4	UV stabilizers as well as antioxidants for hydrocarbon-based products .varying from petrochemicals to **plastics**.
Chem34	1-Cyclohexene-1-carboxylic acid, 4-(1,5-dimethyl-3-oxohexyl)-, methyl ester, [S-(R*,R*)]- CAS: 26462-72-6	not a well-known or widely used chemical.
Chem35	1,2-Propanedione, 1-phenyl-, 2-oxime ǀ CAS: 119-51-7	**flavor and fragrance** materials and pharmaceutical intermediates.
Chem36	1,1'-Biphenyl, 2,2'-diethyl- CAS: 13049-35-9	A benzenoid aromatic compound occurs naturally in coal tar, crude oil, and natural gas. Formerly used as a **fungicide** for citrus crops. An **antimicrobial food preservative** and an **antifungal** agrochemical.
Chem37	Ethanol, 2-(2-butoxyethoxy)- CAS: 112-34-5	**Solvent**/Alcohol/Ether, Glycol Ether.
Chem38	Butylated Hydroxytoluene CAS: 128-37-0	Preservative food additive and antioxidant .
Chem39	Benzophenone CAS: 119-61-9	A photo initiator for Ultra-violet curing applications with inks, imaging, and clear coatings in the printing industry. Prevents UV light from damaging scents and colors in products-perfumes and soaps. *PAN bad actor**
Chem40	Phenol, 4-(1,1-dimethylpropyl)- CAS: 80-46-6	**Microbiocide** Phenol. *PAN bad actor**
Chem41	Phenol, p-tert-butyl- (or positional isomer) CAS: 98-54-4	**Microbiocide**, Phenol .
Chem42	Phenol, 4-chloro-3-methyl-: CAS: 59-50-7	**Microbiocide**, Fungicide, Chlorinated Phenol. *PAN bad actor**
Chem43	Homosalate CAS: 118-56-9	**Insecticide,** Fungicide, Microbiocide, Benzoic acid.
Chem44	Benzyl alcohol CAS: 100-51-6	**Insecticide**, Fungicide.
Chem45	2-Ethylhexyl salicylate CAS: 118-60-5	**Insecticide.**
Chem46	Dibutyl phthalate CAS: 84-74-2	Insect Repellent, **Insecticide**. *PAN bad actor**
Chem47	1,2-Benzenedicarboxylic acid, bis(2-methylpropyl) ester CAS: 84-69-5	Insect Repellent**, Insecticide.**
Chem48	Diethyltoluamide CAS: 134-62-3	**DEET**, Insect Repellent.
Chem49	Cholesterol CAS: 57-88-5	Steroid

Journal of Xenobiotics

Chem50	Caprolactam CAS: 105-60-2	nylon 6 fibers and resins and nylon 6 **polymer** used in synthetic fibers, textile, carpet, and industrial yarn industries.
Chem51	2-Propenoic acid, 3-(4-methoxyphenyl)-, 2-ethylhexyl ester CAS: 5466-77-3	UV absorber and UV filter in sunscreens.
Chem52	Valproic Acid CAS: 99-66-1	Anticonvulsant drug. Also used for treating migraine headaches and manic episodes in bipolar disorder, potentially through increasing gamma-aminobutyric acid (GABA) in the brain. When taken during pregnancy, has also been linked to increased risk for autism. *Pan Bad Actor**
Chem53	2-Pyrrolidinone, 1-methyl- CAS: 872-50-4	Chemical **intermediate**, Electronics production, Food additive, Fuel or fuel additive**, Solvent**
Chem54	Ethanol, 2-(2-ethoxyethoxy)- CAS: 111-90-0	**Solvent**/Adjuvant

*Note: To identify the most toxic set of pesticides, the Pesticide Action Network (PAN) created the term PAN Bad Actor. These pesticides are at least a known or probable carcinogen, a reproductive or developmental toxicants, a neurotoxic cholinesterase inhibitor, a known groundwater contaminant, or a pesticide with high acute toxicity,.
